# Clinical application and outcomes of sentinel node navigation surgery in patients with early gastric cancer

**DOI:** 10.18632/oncotarget.17584

**Published:** 2017-05-03

**Authors:** Takaaki Arigami, Yoshikazu Uenosono, Shigehiro Yanagita, Keishi Okubo, Takashi Kijima, Daisuke Matsushita, Masahiko Amatatsu, Takahiko Hagihara, Naoto Haraguchi, Yuko Mataki, Katsuhiko Ehi, Sumiya Ishigami, Shoji Natsugoe

**Affiliations:** ^1^ Department of Digestive Surgery, Breast and Thyroid Surgery, Kagoshima University Graduate School of Medical and Dental Sciences, Kagoshima, Japan; ^2^ Molecular Frontier Surgery, Kagoshima University Graduate School of Medical and Dental Sciences, Kagoshima, Japan

**Keywords:** sentinel node navigation surgery, basin dissection, sentinel node mapping, outcomes, early gastric cancer

## Abstract

Sentinel node navigation surgery (SNNS) has been recognized as a minimally invasive tool for individualized lymphadenectomy in patients with early gastric cancer (EGC). The aim of this study was to compare clinicopathological factors, adverse events, and clinical outcomes between sentinel node mapping (SNM) and SN dissection (SND) groups and assess the clinical utility of SNNS in patients with EGC. The clinical data of 157 patients with EGC, diagnosed as clinical T1N0M0 with tumors ≤ 40 mm, undergoing SNNS between March 2004 and April 2016 were retrospectively reviewed. Twenty-seven patients were excluded from the analysis. In the remaining 130 patients, 59 and 71 patients underwent standard lymphadenectomy for SNM and SND, respectively. The sentinel node detection rate in the SNM and SND groups was 98.3% (58/59) and 100% (71/71), respectively. Two (3.5%), 15 (25.9%), and 41 (70.7%) patients having sentinel nodes underwent total gastrectomy, proximal gastrectomy (PG), and distal gastrectomy (DG), respectively, in the SNM group. One (1.4%), 5 (7.0%), 10 (14.1%), 39 (54.9%), and 16 (22.5%) patients underwent PG, DG, segmental gastrectomy, local resection, and endoscopic submucosal dissection, respectively, in the SND group. There was no significant difference in postoperative complications between the SNM and SND groups (*P* = 0.781). Survival did not differ between the both groups (*P* = 0.856). The present results suggest that personalized surgery with SND provides technical safety and curability related with a favorable survival outcome in patients with EGC.

## INTRODUCTION

Gastric cancer is one of the most common malignancies in Asia [1]. In particular, the prevalence of early gastric cancer (EGC) among patients with gastric cancer ranges from 50% to 60% in Japan [2, 3]. These findings indicate that the percentage of patients with EGC has increased. Furthermore, surgical treatments including endoscopic approaches currently vary in patients without clinical lymph node metastasis (cN0). Therefore, it is difficult to determine the therapeutic plan for patients with early gastric tumors and cN0.

To date, many investigators have demonstrated the potential utility of sentinel node navigation surgery (SNNS) in patients with EGC who are preoperatively free of lymph node metastasis [4–9]. According to a prospective multicenter trial in Japan, the sentinel node (SN) detection rate and the accuracy of SNNS for metastatic status in 397 patients with clinical T1 (cT1) or T2 (cT2) and cN0 gastric cancer measuring < 4 cm were 97.5% and 99%, respectively [8]. Furthermore, we focused on lymph node micrometastasis and reported that SNNS is a promising surgical tool for patients with cT1N0 gastric cancer, even when lymph node micrometastasis is detectable by molecular approaches, such as immunohistochemistry (IHC) and reverse transcription-polymerase chain reaction (RT-PCR) [5, 6]. Not surprisingly, patients enrolled in these studies underwent curative gastrectomy with standard lymphadenectomy for SN mapping (SNM), and patients treated with individualized gastrectomy based on the SN concept were excluded from these studies [4–9].

Currently, SNNS has actually been performed as a clinical option for personalized treatment in patients with EGC at advanced institutions. Then, SN basin dissection, rather than pick-up method, has been recommended to decrease false-negative cases and perform SNNS safely in the clinical procedure of SN dissection (SND) [4, 8, 9]. Moreover, investigators have reported that a dual-tracer method with radioactive colloids and dyes is favorable for easy detection of and not overlooking sentinel nodes (SNs) [8, 9]. Thus, technical approaches in SNNS have already been established. A few reports have compared postoperative adverse events and clinical outcomes between SNM and SND in patients with cT1N0 gastric cancer undergoing SNNS. Thus, the aim of the present study was to assess surgical safety and oncologic curability in patients receiving individualized treatments with SND as a clinical application of SNNS.

## RESULTS

### Patients’ preoperative characteristics

Table 1 shows the clinicopathological factors at the time of preoperative diagnosis in 59 and 71 patients of the SNM and SND groups, respectively. There were no significant differences in sex, age, tumor location, and histological type between the SNM and SND groups (*P* = 0.278, *P* = 0.597, *P* = 0.367, and *P* = 0.860, respectively). The mean tumor sizes (± SD) in the SNM and SND groups were 25.9 ± 8.9 mm and 14.9 ± 5.9 mm, respectively; preoperative tumor size was significantly larger in the SNM group (*P* < 0.0001). Regarding the depth of tumor invasion, the 59 patients with SNM had 4 cT1a and 55 cT1b tumors. On the other hand, the 71 patients with SND had 26 cT1a and 45 cT1b tumors. Patients with SNM had a significantly higher incidence of cT1b tumors than those with SND (*P* < 0.0001). The majority of patients with cT1a, cT1b1 (tumor invasion is within 0.5 mm of the muscularis mucosae), and tumors ≤ 20 mm was selected as the SND group.

**Table 1:** Patients’ preoperative clinical characteristics by group

Factor	SNM (%)	SND (%)	*P*-value
	(n = 59)	(n = 71)	
Sex			
Male	40 (67.8)	41 (57.8)	0.278
Female	19 (32.2)	30 (42.2)	
Age (mean years)	67.0 ± 9.8	65.3 ± 13.1	0.597
Tumor location			
Upper	15 (25.4)	21 (29.6)	0.367
Middle	27 (45.8)	37 (52.1)	
Lower	17 (28.8)	13 (18.3)	
Tumor size (mm)	25.9 ± 8.9	14.9 ± 5.9	< 0.0001
Depth of tumor invasion			
cT1a	4 (6.8)	26 (36.6)	< 0.0001
cT1b	55 (93.2)	45 (63.4)	
Histological type			
Differentiated	29 (49.2)	33 (46.5)	0.860
Undifferentiated	30 (50.8)	38 (53.5)	

SND: sentinel node dissection; SNM: sentinel node mapping.

### Detection of sentinel nodes

SNs were identified in 58 (98.3%) of 59 patients with SNM. All patients with SND had SNs (SN detection rate: 100%). The mean numbers of SNs (± SD) in the SNM and SND groups were 4.1 ± 2.5 and 4.2 ± 2.2, respectively. There was no significant difference in the number of SNs between the SNM and SND groups (*P* = 0.443).

### Metastatic status in sentinel nodes and non-sentinel nodes

**Figure 1:**
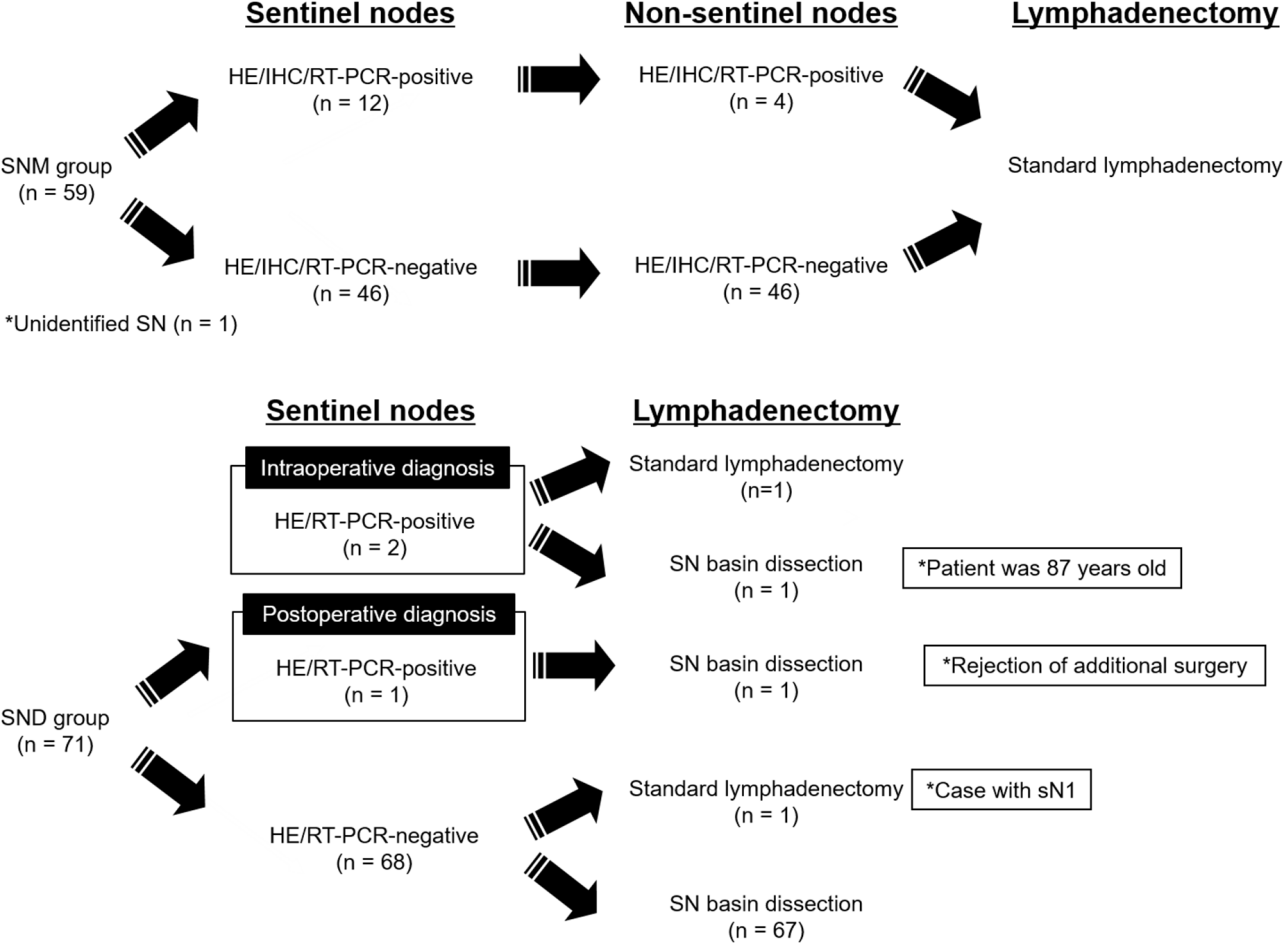
Metastatic status in sentinel nodes and non-sentinel nodes

Among the 58 patients with identified SNs in the SNM group, 12 had lymph node metastasis in the SNs. Of these, 4 had lymph node metastasis in both SNs and non-SNs (Figure 1). None of the remaining 46 patients without metastatic SNs had lymph node metastasis in non-SNs. Accordingly, the false-negative and accuracy rates in the SNM group were 0% and 100%, respectively.

Among the 71 patients with SND, 2 patients had metastatic nodes by intraoperative diagnosis of SNs (Figure 1). Therefore, the surgical procedure was converted to D2 gastrectomy in one of these patients with metastatic SNs. After surgery, pathological re-assessments by hematoxylin-eosin (HE) staining demonstrated that one patient with SND had a metastatic node among the SNs. Since one patient in the SND group had a gross metastatic node (sN1) during surgery of the remaining 68 patients without metastatic SNs, modified D2 gastrectomy was performed in this patient. Finally, individualized surgical or endoscopic treatments with SND were performed in 67 patients without metastatic SNs (Figure 1).

### Surgical procedures and dissected lymph nodes

In 58 patients of the SNM group with identified SNs, 41 (70.7%), 15 (25.9%), and 2 (3.5%) patients underwent distal gastrectomy (DG), proximal gastrectomy (PG), and total gastrectomy (TG), respectively (Table 2). In the SND group, 5 (7.0%), 1 (1.4%), 10 (14.1%), 39 (54.9%), and 16 (22.5%) patients underwent DG, PG, segmental gastrectomy (SG), local resection, and endoscopic submucosal dissection (ESD), respectively (Table 2). In the SND group, one patient with metastatic SNs detected by intraoperative diagnosis did not undergo standard lymphadenectomy due to advanced age (87 years old). Moreover, one patient with a metastatic SN identified by postoperative HE staining refused an additional gastrectomy with lymphadenectomy in the SND group.

**Table 2:** Surgical procedures

	Surgical procedure (%)					
	DG	PG	TG	SG	LR	ESD
SNM (n = 58)	41 (70.7)	15 (25.9)	2 (3.5)	0 (0.0)	0 (0.0)	0 (0.0)
SND (n = 71)	5 (7.0)	1 (1.4)	0 (0.0)	10 (14.1)	39 (54.9)	16 (22.5)

DG: distal gastrectomy; ESD: endoscopic submucosal dissection; LR: local resection; PG: proximal gastrectomy; SG: segmental gastrectomy; SND: sentinel node dissection; SNM: sentinel node mapping; TG: total gastrectomy.

The mean numbers of dissected lymph nodes (± SD) in the SNM and SND groups were 27.4 ± 13.1 and 13.3 ± 10.1, respectively. Patients with SND had significantly fewer dissected lymph nodes than those with SNM (*P* < 0.0001).

### Pathological assessment of primary tumors

Table 3 shows the pathological findings based on HE staining of the primary tumors of patients in the SNM and SND groups. The mean tumor sizes (± SD) in the SNM and SND groups were 31.3 ± 18.1 mm and 16.4 ± 9.7 mm, respectively. Consequently, SNM patients had significantly larger primary tumors than SND patients (*P* < 0.0001). In the SNM group of 59 patients, there were 29 pT1a (49.2%), 26 pT1b (44.1%), 2 pT2 (3.4%), and 2 pT3 (3.4%) tumors. In the SND group of 71 patients, there were 50 pT1a (70.4%), 19 pT1b (26.8%), and 2 pT2 (2.8%) tumors. Furthermore, lymphatic invasion in the SNM and SND groups was identified in 12 of 59 and 7 of 71 patients, respectively. Eight and six patients had venous invasion in the SNM and SND groups, respectively. There were no significant differences in the depth of tumor invasion, lymphatic invasion, and venous invasion between the two groups (*P* = 0.054, *P* = 0.134, and *P* = 0.403, respectively).

**Table 3:** Pathological findings of primary tumors

Factor	SNM (%)	SND (%)	*P*-value
	(n = 59)	(n = 71)	
Tumor size (mm)	31.3 ± 18.1	16.4 ± 9.7	< 0.0001
Depth of tumor invasion			
pT1a	29 (49.2)	50 (70.4)	0.054
pT1b	26 (44.1)	19 (26.8)	
pT2	2 (3.4)	2 (2.8)	
pT3	2 (3.4)	0 (0.0)	
Lymphatic invasion			
Positive	12 (20.3)	7 (9.9)	0.134
Negative	47 (79.7)	64 (90.1)	
Venous invasion			
Positive	8 (13.6)	6 (8.5)	0.403
Negative	51 (86.4)	65 (91.5)	

SND: sentinel node dissection; SNM: sentinel node mapping.

### Postoperative complications

None of the patients had serious allergic reactions associated with the injection of ^99m^Tc-tin colloid, isosulfan blue, or indocyanine green (ICG). Table 4 shows postoperative complications of grade II or higher of the Clavien-Dindo classification in the SNM and SND groups; 7 (11.9%) and 7 (9.9%) patients had postoperative complications in the SNM and SND groups, respectively. No significant difference in the incidence of postoperative complications was observed between the two groups (*P* = 0.781).

**Table 4:** Postoperative complications

Adverse event	SNM (%)	SND (%)
	(n = 59)	(n = 71)
Surgical site infection	1 (1.7)	2 (2.8)
Anastomotic leakage or perforation	1 (1.7)	2 (2.8)
Delayed gastric emptying	1 (1.7)	1 (1.4)
Anastomotic stenosis	0 (0.0)	1 (1.4)
Pneumonia	2 (3.4)	0 (0.0)
Cerebral infarction	1 (1.7)	0 (0.0)
Deep vein thrombosis	1 (1.7)	1 (1.4)

SND: sentinel node dissection; SNM: sentinel node mapping.

### Survival

**Figure 2:**
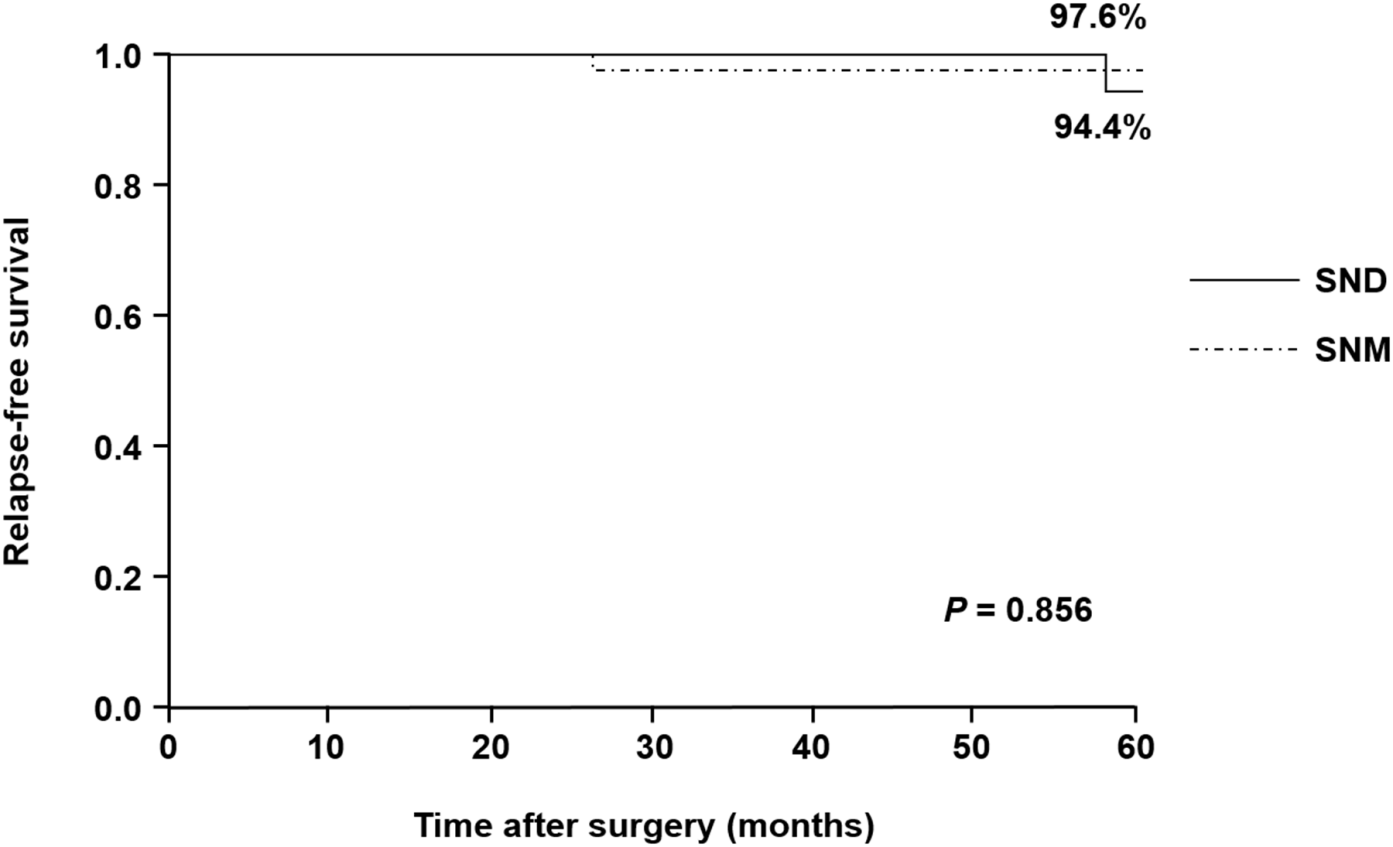
Relapse-free survival curves in the SNM and SND groups The 5-year relapse-free survival rates in the SNM and SND groups are 97.6% and 94.4%, respectively.

The 5-year relapse-free survival (RFS) rates in the SNM and SND groups were 97.6% and 94.4%, respectively (Figure 2). None of the patients had disease recurrence in either group. No significant differences were observed in RFS between the SNM and SND groups (*P* = 0.856).

## DISCUSSION

To assess the surgical safety and oncologic curability of patients with EGC undergoing SND, postoperative complications and prognosis were compared between SNM and SND groups in the present study. Many investigators have reported that SNNS is applicable to patients with cT1N0 EGC [4–9]. However, most of these reports were based on results obtained from patients with EGC undergoing conventional gastrectomy with standard lymphadenectomy for SNM [4–9]. To the best of our knowledge, this is the first study to evaluate the validity of SND as a clinical application of SNNS in patients with cT1N0 EGC.

In this study, patients with cT1N0 gastric cancer measuring ≤ 40 mm in tumor diameter were enrolled retrospectively. According to a prospective multicenter trial in Japan, the false-negative rate in patients with cT1 and cT2 tumors was 0.9% and 1.9%, respectively [8]. This result indicates that patients with cT2 tumors had a higher false-negative rate than those with cT1 tumors. Finally, this multicenter trial concluded that SNNS should be limited to patients with cT1N0 tumors. Therefore, patients with cT2N0 tumors were excluded from the present study. It is clinically important to determine the indication for SNNS in preoperative management to prevent lymph node recurrence. In the present study, there were significant differences in preoperative tumor size and the clinical depth of tumor invasion between the SNM and SND groups (*P* < 0.0001). In the initial phase for performing SND, we had limited the indication for SND to patients with mucosal tumors (cT1a). Accordingly, our policy may have resulted in these findings.

A dual-tracer method using radioactive colloids and dyes was basically used in both the SND and SNM groups. This dual-tracer procedure has been recommended to increase the SN detection rate and decrease the false-negative rate in SNNS [7–9]. Although SNs were detected by RI method alone in 41 patients (31.5%), these patients were involved in the initial phase of SNNS. Unfortunately, SNs were not detected in 1 of 59 patients with SNM in the present study. On the other hand, SNs were identified in all patients with SND. An ICG fluorescence imaging system was used in 28 (39.4%) of 71 patients with SND. Consequently, the introduction of this novel imaging system may have a great effect on the difference in the SN detection rate. Several investigators have demonstrated the clinical utility of the ICG fluorescence imaging approach for SN detection in patients with several malignant neoplasms, including gastric cancer [10–13]. Compared to a conventional dye approach under visual recognition, the ICG fluorescence image-guided method could clearly visualize SNs and afferent lymphatic vessels from primary tumor sites. In future SNNS, an ICG fluorescence imaging system would be seen as an indispensable tool for detecting SNs.

In the present study, no significant differences were seen in the mean numbers of SNs between the SNM and SND groups. However, the numbers of dissected lymph nodes were significantly fewer in SND than in SNM patients (*P* < 0.0001). These results indicate the surgical features of individualized lymphadenectomy based on the SN concept in the SND group. Interestingly, the majority (91.5%) of patients with SND underwent functional-preserving gastrectomy, such as SG, local resection, and ESD. According to the criteria of the Japanese Gastric Cancer Treatment Guidelines 2010 (ver. 3), the expanded indication of ESD is described as an investigational treatment in patients with early gastric cancer [23]. Therefore, we suggest that SNNS by endoscopic procedures is clinically applicable to patients meeting the expanded indication of ESD at the moment. Although postgastrectomy syndrome and quality of life (QOL) in the SNM and SND groups were not assessed in this study, several investigators have suggested that pylorus-preserving gastrectomy and local resection are promising surgical procedures to reduce postoperative disorders and improve QOL in patients with gastric cancer [14–16]. In the near future, prospective multicenter trials would be needed to establish the clinical benefit of functional-preserving gastrectomy in comparison with conventional gastrectomy, such as TG, DG, and PG.

Currently, intraoperative diagnosis for lymph node metastasis is commonly assessed by pathological HE staining in patients with gastric cancer. We have previously demonstrated the clinical importance of lymph node micrometastasis in patients with gastric cancer [17, 18]. In the present study, reverse transcription-polymerase chain reaction (RT-PCR) assays were used to detect lymph node micrometastases. Although none of the patients with SND had micrometastatic nodes identified by RT-PCR in pathologically negative SNs, RT-PCR could recognize a metastatic node in at least pathologically positive nodes confirmed by HE staining. Since the clinical significance of lymph node micrometastasis or isolated tumor cells remains controversial in patients with gastric cancer at present, it is important to assess lymph node metastasis including micrometastasis to avoiding disease recurrence after SNNS and provide safe SNNS. Therefore, we suggest that micrometastatic nodes determined by IHC and RT-PCR during operation should be removed in the intraoperative management for the reduction of lymphadenectomy, including SNNS.

According to previously published reports, postoperative morbidity and mortality rates after D2 gastrectomy were 12.9–20.9% and 0–0.8%, respectively [19, 20]. Although the incidence of postoperative complications in the SND group of the present study was 9.9%, the mortality rate was zero. These findings suggest that SNNS has been established as a personalized treatment providing technical safety, compared with standard gastrectomy, in patients with EGC. Niihara et al. reported that 5-year RFS in 380 patients with cT1N0 or cT2N0 gastric cancer who underwent SNM was 96.2% [21]. This study showed that 5-year RFS rates in patients with SNM and SND were 97.6% and 94.4%, respectively. These results indicate that personalized surgery based on the SN concept could assure oncological curability from the perspective of survival.

There were several limitations to the present study. This preliminary study was based on retrospective data designed by the small patient sample (n = 130) in a single institution. Therefore, this non-prospective analysis may have resulted in a selection bias between the SNM and SND groups. Then, this bias might have an impact on several results in the present study. Furthermore, the median follow-up period was only 37 months. Accordingly, larger prospective studies based on long follow-up period are essential in order to strengthen the present results.

In conclusion, it was demonstrated that SNNS could provide technical safety and oncological curability in patients with cT1N0 EGC. Currently, laparoscopic and endoscopic cooperative surgery (LECS) has been focused on as a novel less-invasive procedure in patients with upper gastrointestinal tumors, including gastric cancer [22]. In the future, LECS will contribute greatly to the development of SNNS for patients with EGC.

## MATERIALS AND METHODS

### Patients

**Figure 3:**
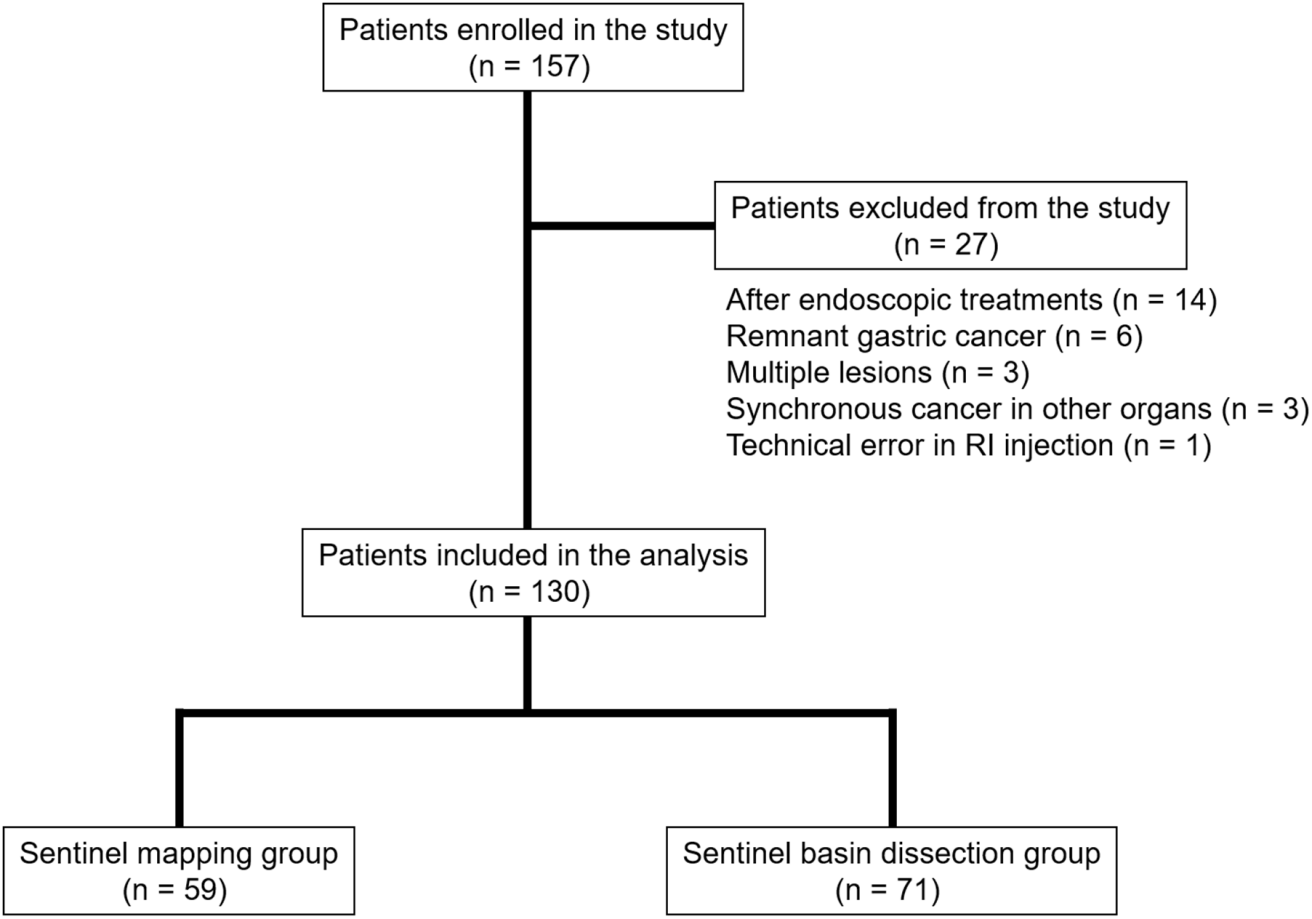
Patient enrollment and surgical treatments Of the 130 enrolled patients, 59 and 71 underwent standard lymphadenectomy for sentinel node mapping (SNM) and sentinel node dissection (SND), respectively.

Patients with EGC, diagnosed as cT1N0M0 with tumors ≤ 40 mm, were retrospectively reviewed. All patients were assessed by esophagogastroduodenoscopy, endoscopic ultrasonography, and computed tomography before SNNS. Then, all patients were pathologically confirmed to have adenocarcinoma of the stomach by endoscopic biopsy. Patients with gastric tumors for which endoscopic treatment was considered indicated based on the criteria of the Japanese Gastric Cancer Treatment Guidelines 2010 (ver. 3) established by the Japanese Gastric Cancer Association were excluded from the present study [23]. In total, 157 consecutive patients were enrolled between March 2004 and April 2016. Twenty-seven patients were excluded from this analysis, including 14 patients after endoscopic treatments, 6 patients with remnant gastric cancer, 3 patients with multiple lesions, 3 patients with synchronous cancer in other organs, and one patient with a technical error of radioisotope (RI) injection. In the remaining 130 enrolled patients, 59 and 71 patients underwent standard lymphadenectomy for SNM and SND, respectively, at Kagoshima University Hospital (Figure 3). Surgically resected specimens were classified on the basis of the Japanese Classification of Gastric Carcinoma (3rd English edition) [24]. Each two patients in the SNM and SND groups received adjuvant chemotherapy with S-1. The median follow-up period was 37 months (range, 1–142 months).

The Ethics Committee of Kagoshima University approved the study, and all patients provided their written, informed consent to participate in all procedures associated with this study.

### Sentinel node navigation surgery

One day before surgery, 4 mCi (2 ml) of ^99m^technetium (^99m^Tc)-tin colloid were endoscopically injected into the submucosa of the gastric wall at 4 sites (0.5 ml each) around the tumor using a disposable 23-gauge needle (MAJ–75, Olympus, Japan) in all patients. Furthermore, 1% isosulfan blue or ICG was used as a dye tracer in 89 of 130 patients, and these dyes were similarly injected before surgery. Accordingly, SNs were identified by RI method alone in the remaining 41 patients. During surgery, SN detection was based on the uptake of RI using Navigator GPS (TYCO HEALTHCARE, Ltd., Tokyo, Japan) and visual assessments by dyes. In the SND group after June 2012, a laparoscopic ICG fluorescence imaging system (Olympus, Japan) was introduced. In surgical procedures using a laparoscopic ICG fluorescence system, stained lymph nodes were identified and marked by clip under *in vivo* imaging. Then, lymphatic basin dissection was performed.

Although 18 patients underwent SNNS by pick-up method in the SND group before January 2008, the remaining 53 patients underwent selective lymphadenectomy to dissect lymphatic basins including lymph nodes and afferent lymphatic vessels stained by dye in surgical procedures for SND. Pick-up method was adopted in the beginning phase of SNNS. Subsequently, we had adopted lymphatic basin dissection when performing SNNS. On the other hand, standard lymphadenectomy was performed based on the Japanese Gastric Cancer Treatment Guidelines 2010 (ver. 3) after intraoperative SN detection in the SNM group [23]. After harvesting all dissected lymph nodes in both the SNM and SND groups, RI uptake was counted in all dissected lymph nodes. Lymph nodes that absorbed 10 times more RI than the background level were defined as SNs in the present study.

### Lymph node processing

Lymph node metastasis in the SNM group was assessed by HE staining, IHC using anti-human cytokeratin (CK) monoclonal antibody (mAb), and RT-PCR. To detect lymph node micrometastasis, IHC and/or RT-PCR assay was conducted in 47 of 59 patients with SNM. Then, RT-PCR assay was postoperatively performed in the SNM group. Since all patients with SNM underwent standard lymphadenectomy, we did not routinely perform these assays for detecting lymph node micrometastasis.

All SNs in the SND group were cut into three blocks, and two blocks were used intraoperatively for HE staining and RT-PCR assay. A tissue block for HE staining was sliced into multiple sections at the plane of the largest dimension. RT-PCR assay was conducted in 42 of 71 patients with SND. The remaining block was fixed in 10% formaldehyde, embedded in paraffin, and then cut into 3-μm-thick sections. These sections were used for pathological re-assessment by HE staining after surgery. In this study, lymph nodes that had positive status by at least one diagnostic tool were defined as metastatic nodes.

### RT-PCR assay

SNs were homogenized, and total RNA was purified using the RNA Sample Preparation Kit (Veridex LLC, Huntingdon Valley, PA). Samples were analyzed using a prototype kit run on the Cepheid SmartCycler system (Cepheid, Sunnyvale, CA). This system has been shown to have clinical utility to finalize reverse transcription of the complementary DNA (cDNA) and amplification of the cDNA in one step within approximately 40 minutes, as previously described [25]. Moreover, this kit was designed to detect mRNA expressions of carcinoembryonic antigen (CEA), cytokeratin 19 (CK 19), and porphobilinogen deaminase as an internal control. The cutoff values of the threshold cycle (Ct) in CEA and CK 19 were set at 38 Ct and 37 Ct, respectively, as previously described [25].

### Immunohistochemistry

CK AE1/AE3 mAb (DAKO Corporation, Carpinteria, CA) was used for IHC staining. The 3-μm-thick paraffin-embedded archival tissue (PEAT) sections were deparaffinized in xylene and rehydrated in ethanol, and then endogenous peroxidase activity was blocked by 5-min incubation in methanol containing 3% hydrogen peroxide. The sections were then immersed in proteinase K (DAKO Corporation) to activate the antigen and incubated with CK mAb diluted 1:200 for 30 min. The sections were washed with phosphate-buffered saline (PBS), and CK was stained using the ABC method (Vectastain ABC kit, Vector Laboratories, Inc., Burlingame, CA) and visualized using diaminobenzidine tetrahydrochloride. PEAT sections of normal gastric mucosa were used as positive controls. Negative controls were treated with PBS without primary antibody under the same conditions.

### Statistical analysis

Differences in categorical clinicopathological factors between the SNM and SND groups were assessed using the chi-square and Fisher’s exact tests. Differences in age, tumor size, numbers of dissected lymph nodes, and numbers of SNs between the SNM and SND groups were evaluated using the Wilcoxon rank sum test. RFS was determined from the date of SNNS to the date of the first evidence of recurrence or death. Survival curves were constructed using the Kaplan-Meier method, and differences were determined using the log-rank test. All data were statistically analyzed using SAS statistical software (SAS Institute Inc., Cary, NC). A *P*-value of < 0.05 was considered significant.
